# Self-perceived knowledge, attitude, and practice of evidence-based medicine before and after training among healthcare workers in Taizhou, China

**DOI:** 10.1186/s12909-024-05678-7

**Published:** 2024-06-27

**Authors:** Chengwen Luo, Mei-Xian Zhang, Yu-pei Yang, Tao-Hsin Tung

**Affiliations:** 1 Evidence-based Medicine Center, Taizhou Hospital of Zhejiang Province affiliated to Wenzhou Medical University, Linhai, Zhejiang China; 2 Department of Hematology, Taizhou Hospital of Zhejiang Province affiliated to Wenzhou Medical University, Linhai, China; 3 Taizhou Institute of Medicine, Health and New Drug Clinical Research, Taizhou, China

**Keywords:** Healthcare workers, Evidence-based medicine, Knowledge, Attitude, Practice

## Abstract

**Background:**

Evidence-based medicine (EBM) is the combination of the best research evidence with our clinical expertise, specific situations, and the unique values of our patients. It is essential to evaluate the effectiveness of EBM training for healthcare workers (HCWs).

**Objectives:**

This study aims to assess the impact of EBM training on HCWs’ knowledge, attitude, and practice (KAP) related to EBM.

**Methods:**

A self-reported online survey was carried out to investigate KAP related to EBM among HCWs at a tertiary hospital in Taizhou, China. HCWs participated in EBM training on 9 and 10 September 2023. The questionnaire survey was conducted to understand KAP related to EBM before and after the training, and to compare and analyze the results before and after the training. The R software (version 4.1.0) was used to analyze data.

**Results:**

Sixty-four HCWs completed the survey with a response rate of 52.5% (64/122). The overall average scores of KAP related to EBM before training were 55.3, 63.0, and 34.5, respectively, and 56.9, 66.5, and 34.7 were the scores of KAP after training. HCWs’ scores of knowledge (*P* = 0.033) and attitude (*P* < 0.001) related to EBM improved significantly after the training.

**Conclusion:**

This study implied that EBM training may improve the knowledge and attitude of HCWs, and its teaching effect is considerable.

**Supplementary Information:**

The online version contains supplementary material available at 10.1186/s12909-024-05678-7.

## Introduction

Evidence-based medicine (EBM) is defined as “the careful, explicit, and intelligent use of the best evidence in making decisions about patient care” [1]. In recent decades, the concept of EBM has penetrated clinical, nursing, and other medical and health fields, and has become one of the disciplines advocated by international clinical medicine [2]. It is also known that EBM can help healthcare workers (HCWs) integrate the latest study evidence into their clinical practices [3]. It is important for HCWs to adopt EBM to provide cost-effective and safe care, as combining individual clinical expertise with the best currently available external clinical evidence and patient values facilitates the clinical decision-making process. The systematic reviews performed by the Cochrane Library denote the gold standard for EBM practice [4].

EBM has been adopted in many medical care professions such as clinical diagnosis, occupational therapy, and nursing [5]. To practice EBM, individuals should develop clear clinical study questions, search for study information, critically evaluate the study evidence obtained, determine the applicability of the evidence to patients’ care, and evaluate the overall performance. However, implementing evidence-based practice in daily clinical practice is a complex and challenging process [6, 7]. The previous study indicated that the knowledge and attitude related to EBM were associated with the practice of EBM [8].

In recent decades, a large number of EBM training approaches have been studied to encourage the application of EBM in medical practice [9–11]. The purpose of EBM training is to enable the subjects to have the concept of EBM in medical practices, and to establish a new medical decision-making model that integrates the best study evidence, scientific clinical experience, and patients’ wishes. The common training methods were varied such as research courses, problem-based, small groups, online learning, or distance education [12]. For example, a well-designed EBM course was established for undergraduate medical education in the previous training [13]. In addition, to improve the skills of EBM, a problem-based method was presented, whereby students presented a focused clinical question and gave a presentation through contact with real patients during their recent clinical practice [14, 15].

Previous research mainly focused on the effectiveness of EBM training for medical students, while only a few studies concentrated on the knowledge, attitude, and practice (KAP) of EBM among HCWs in China. To ensure that healthcare users can enjoy good medical care in the future, it is necessary to effectively incorporate EBM-related KAP into educational programs for HCWs. The aim of this study was to compare HCWs’ KAP changes before and after EBM training, so as to evaluate the effect of EBM training and provide scientific suggestions for EBM teaching and reform.

## Methods

### Ethical approval

This research was waived from informed consent by the Ethical Review Committee of Taizhou Hospital, Zhejiang Province, China and the study was approved by the Ethical Review Committee of Taizhou Hospital, Zhejiang Province, China (Approval number: K20231021). Information about all participants was recorded anonymously. All procedures were conducted according to the guidelines of our institutional Ethics Committee and in compliance with the principles of the Declaration of Helsinki.

### Study design and participants

This study was a cross-sectional experimental investigation along with before and after training assessment. The target population in the EBM training was HCWs at a medical center in Taizhou, China. This group of people want to continue to improve their research capabilities. The goal of this training was to improve the awareness and attitude of healthcare professionals towards EBM, so as to better practice EBM. We designed an online survey to evaluate their KAP before and after the training, respectively. The largest online survey platform in China named Wen-Juan-Xing platform (Changsha Ranxing Information Technology Co., Ltd., Hunan, China) was used to collect data about KAP related to EBM before and after the training. HCWs received the questionnaire via WeChat or e-mail, and they responded to the questionnaire by accessing the Uniform Resource Location (URL) or scanning a Quick Response (QR) code on their cell phones or computers.

### Educators

Theoretical teaching and practical operation were provided by four faculty members who were experts on EBM and biostatistics.

### Intervention

To improve the capacity of EBM among HCWs, it is important to understand the basic approaches of epidemiology and biostatistics, as these skills are commonly used in conducting, analyzing, and reporting medical studies [16, 17]. In this study, the educational intervention consisted of a module of lessons centered on the theme of EBM course was conducted at the Taizhou Hospital of Zhejiang Province, Taizhou, China. This teaching carried out a 2-day EBM training course from September 9 to 10, 2023, which consisted of theoretical teaching and practical operation.

**Fig. 1:**
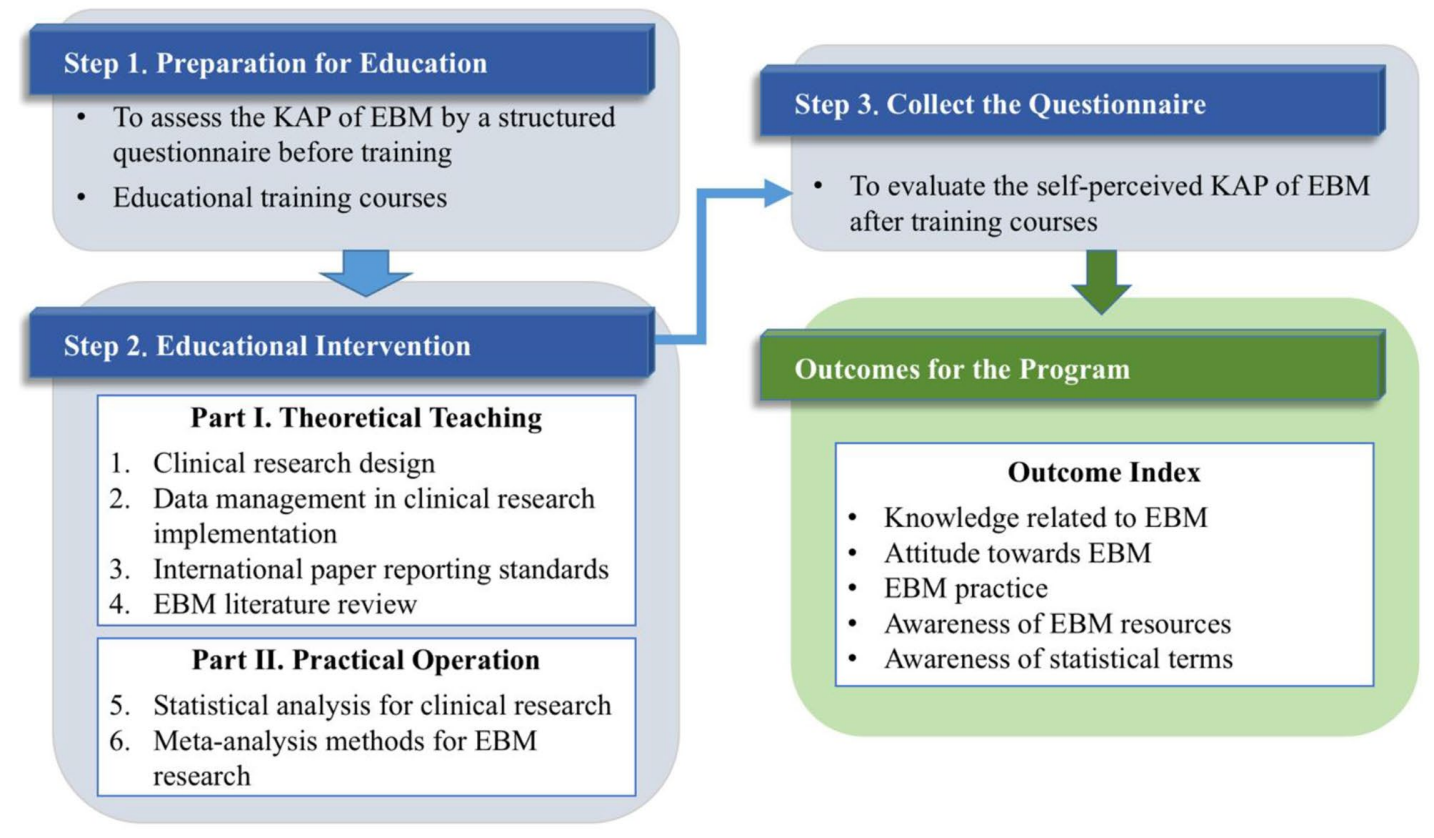
Flowchart of the EBM education training intervention for healthcare workers

The main contents of the training courses included the following aspects. (1) Clinical research design, including condensing clinical science problems, designing research protocols, determining observation indicators, selecting research objects, estimating sample size, and designing statistical analysis protocols; (2) Data management in clinical research implementation, including case report form (CRF), database design, data entry and sorting; (3) International paper reporting standards; (4) EBM literature review, including the concept, method, and quality level of EBM literature review; (5) Statistical analysis for clinical research, including cross-sectional study, case-control study, cohort study, mediation analysis, path analysis, and structural equation; (6) Meta-analysis methods for EBM research. Among the training courses, (1)–(4) were theoretical teaching and (5)–(6) combined with practical operation, based on SPSS and RevMan software. The details of course programs and training schedules were presented in Table 1. Flowchart of the EBM education training intervention for HCWs could be found in Fig. 1.

**Table 1:** Training details

Time	Topic	Objective (Participants will be able to)	Content	Training method
8:00–8:30 am 9/9/2023	Answer the questionnaire before training			
8:30 − 12:00 am 9/9/2023	Clinical research design	• Condense clinical science questions • Name different types of clinical research design • Design clinical study protocol	The content of this lecture included condensing clinical science problems, designing research protocols, determining observation indicators, selecting research objects, and estimating sample size.	Theoretical teaching
13:30 − 15:30 pm 9/9/2023	Data management in clinical research implementation	• Understand and design the case report form • Lists common data management tools • Understand the process of data management	This part included case report form (CRF), database design, and data entry and sorting.	Theoretical teaching
15:30 − 17:30 pm 9/9/2023	International paper reporting standards	• Understand the basic information of international standards for paper reporting • Know how to write a paper • Learn to respond to review comments	The course content included international standard for paper reporting and how does peer review evaluate the science of research.	Theoretical teaching
8:00–10:00 am 9/10/2023	EBM literature review	• Understand the concept of literature review in EBM • List the common evaluation tools • Know how to rate quality	This lecture concentrated on the concept of literature review in EBM, evaluation method, and quality grade.	Theoretical teaching
10:00–12:00 am & 13:30 − 15:00 pm 9/10/2023	Statistical analysis for clinical research	• Master common statistical analysis methods • List the concepts used in the evaluation of statistical significance (such as effectiveness, *p* value, confidence interval) • Learn how to use SPSS software for data analysis	Statistic class included cross-sectional study, case-control study, cohort study, mediation analysis, and structural equation model.	Practical operation (SPSS software)
15:00–17:00 pm 9/10/2023	Meta-analysis methods for EBM research	• Understand the methods for meta-analysis • Summarize the risk of bias • Learn how use RevMan software	Introduction of meta-analysis method, risk of bias, drawing forest map and funnel map.	Practical operation (RevMan software)
17:00–17:30 pm 9/10/2023	Answer the questionnaire after training			

### Study tool

The main contents of the questionnaire contained basic demographic information, KAP related to EBM, EBM resources, and statistical terms based on the previous research [8, 18, 19]. In this study, the survey mainly includes the following aspects. (1) Socio-demographic information: including age, sex, education, occupation, professional technical title, total service years, and previously undergone training courses in EBM. (2) Knowledge related to EBM. Knowledge of EBM was evaluated by 15 items with 5 optional answers: “Strongly agree”, “Agree”, “Neutral”, “Disagree” or “Strongly disagree”. (3) Attitudes toward EBM. Attitudes toward EBM were measured by 17 items and assessed similarly. (4) EBM practices: This section consisted of 11 items. Five possible answers were used to evaluate each item statement: “Always”, “Often”, “Sometimes”, “Seldom”, or “Never”. (5) Knowledge and use of common resources in EBM. (6) Knowledge and use of common statistical terms in EBM. The details could be found in the Supplementary Material.

### Measures

All data collected were extracted directly from the Wen-Juan-Xing platform into an MS Excel spreadsheet and coded appropriately to make them suitable for statistical testing. Each item was scored based on the Likert scale method. Take knowledge related to EBM as an example, the 5-point Likert scale was adopted to give 1 ~ 5 points for each item from strongly disagree, disagree, neutral, agree, and strongly agree, respectively. Of note, reverse scoring was used for negatively worded items. In total, scores of 15 items of EBM knowledge were added up in a range of 15 ~ 75 points. In addition, the scores of EBM attitudes were calculated similarly with a range of 17 ~ 85. For EBM practice, the 5-point Likert scale was also used to give 1 ~ 5 points for each item from always, often, sometimes, seldom, and never, respectively. The total score was generated by taking the sum of all items and the range score was 11 ~ 55. A higher score indicated a higher level of EBM knowledge, attitude, or practice.

### Statistical analysis

Categorical variables for basic demographic information were reported in frequency and percentage. To compare the performance related to EBM before and after the training, the Chi-square test was adopted. The descriptive statistics for continuous variables were also calculated, including mean values and standard deviations. The Kolmogorov-Smirnov test was used to assess the normality of quantitative variables. For normally distributed variables, the difference in KAP scores related to EBM before and after the training was compared using the t-test. While the Wilcoxon signed-rank test was used for non-normally distributed variables. Variables were considered statistically significant with a *p*-value < 0.05. Statistical calculations were performed via R software (version 4.1.0).

## Results

### Basic characteristics

**Fig. 2:**
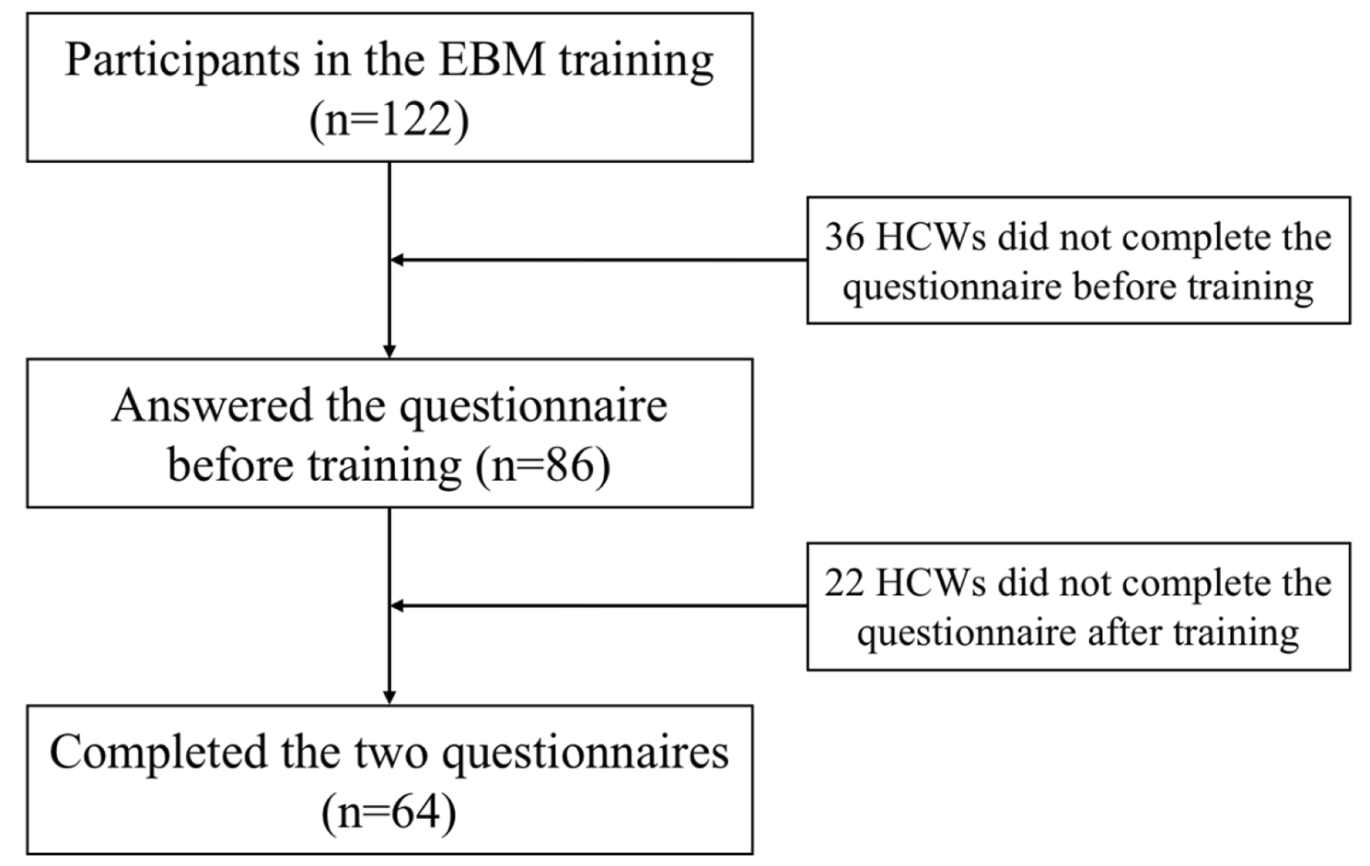
Flowchart of the study participants

A total of 122 HCWs applied to attend the EBM training. Among them, 86 HCWs answered the questionnaire before the training. Finally, sixty-four HCWs completed the two questionnaires, with a response rate of 52.5% (64/122) (Fig. 2).

The basic characteristics were presented in Table 2. The median age of participants was 35 years old and those who were older than 35 accounted for 48.4%. More than half the subjects (70.3%) were females. Respondents who had bachelor’s degrees and postgraduate degrees accounted for 64.1% and 35.9%, respectively. Subjects’ occupational categories include doctor (19, 29.7%), nurse (26, 40.6%), medical technician (13, 20.3%), and administration (6, 9.4%). HCWs who obtained professional titles including primary grade or below, medium grade, and senior grade accounted for 20.3%, 57.8%, and 21.9%, respectively. 57.8%% of the participants had more than 10 total service years. Most of the HCWs (64.1%) had not undergone any training courses in EBM previously.

**Table 2:** Basic characteristics (*n* = 64)

Variable	Category	*n* (%)
Age (years)		
	≤ 35	33 (51.6)
	> 35	31 (48.4)
Gender		
	Male	19 (29.7)
	Female	45 (70.3)
Education		
	Bachelor degree	41 (64.1)
	Postgraduate degree	23 (35.9)
Occupation		
	Doctor	19 (29.7)
	Nurse	26 (40.6)
	Medical technician	13 (20.3)
	Administration	6 (9.4)
Professional titles		
	Primary grade or below	13 (20.3)
	Medium grade	37 (57.8)
	Senior grade	14 (21.9)
Total service time (years)		
	≤ 10	27 (42.2)
	> 10	37 (57.8)
Previously undergone any training courses in EBM		
	Yes	23 (35.9)
	No	41 (64.1)

### Knowledge about EBM

In total, the mean total scores for the knowledge of EBM before and after training were 55.3 and 56.9, respectively (Table 3). Compared with the score before training, HCWs obtained a significantly higher average score after training (*P* = 0.033). The average scores for each statement of the knowledge domain before and after training ranged from 2.4 to 4.4 and 2.7 to 4.6, respectively. There were significant differences between the mean scores of EBM knowledge before and after training in the statements K4 and K13. Take statement K4 for example, the mean (sd) of EBM knowledge before and after training was 2.9 (1.0) and 3.3 (1.2), respectively. For the statement K13, the mean (sd) of EBM knowledge before and after training was 4.0 (0.6) and 4.2 (0.7), respectively. From the descriptive statistics of the level of HCWs’ knowledge about EBM before training, we found that the top three levels were awarded to the statements K9, K12, and K1. While the lower three levels were awarded to the statements K10, K3, and K4. In addition, for the scores of knowledge about EBM after training, the top three were K9, K6, and K8, while the lower three levels were K10, K7, and K3.

**Table 3:** Participants’ knowledge of EBM

Item	Description	Before		After		*P*
		Mean (sd)	Level	Mean (sd)	Level	
K1	Evidence-based medicine involves the process of critically appraising research findings as to the basis for clinical decisions.	4.3(0.8)	3	4.1(0.9)	7	0.657
K2	Evidence-based medicine focuses on the best current available research without considering clinical experience.	3.6(1.2)	10	3.8(1.1)	10	0.145
K3	Evidence-based medicine is suitable for making decisions about the care of patients rather than for policymaking.	2.9(1.3)	14	3.0(1.1)	13	0.382
K4	Patients’ preferences should be prioritized over clinicians’ preferences in making clinical decisions.	2.9(1.0)	13	3.3(1.2)	12	**0.021**
K5	Evidence-based medicine improves clinical management by using evidence from meta-analysis only.	3.4(1.2)	11	3.6(1.3)	11	0.117
K6	Evidence-based medicine does not help to promote self -directed learning.	4.2(1.0)	5	4.3(0.7)	2	0.287
K7	Meta-analysis is superior to case-control studies in evidence-based medicine.	3.1(1.1)	12	2.8(1.2)	14	0.900
K8	Four essential components structured in the PICO format (Patient or problem, Intervention, Comparison, Outcome) will make a good clinical question.	4.2(0.7)	4	4.3(0.7)	3	0.096
K9	Evidence-based medicine improves clinicians’ understanding of research methodology.	4.4(0.6)	1	4.6(0.6)	1	0.053
K10	Clinicians who practice evidence-based medicine become less critical in using data in systematic reviews.	2.4(1.0)	15	2.7(1.2)	15	0.094
K11	Evidence-based medicine can be practiced in situations where there is doubt about any aspect of clinical management.	3.7(1.0)	9	3.8(0.9)	9	0.206
K12	Improving access to summaries of evidence is appropriate to encourage evidence-based practice.	4.3(0.6)	2	4.3(0.6)	4	0.388
K13	The increasing number of systematic reviews that are applicable to general practice can be found in the Cochrane Library.	4.0(0.6)	7	4.2(0.7)	5	**0.029**
K14	Difficulty in understanding statistical terms is the major setback in applying evidence-based medicine.	3.9(0.9)	8	3.9(0.7)	8	0.763
K15	Application of evidence-based practice is cost-effective to the healthcare system.	4.1(0.6)	6	4.2(0.7)	6	0.361
Total		55.3(5.2)		56.9(5.5)		**0.033**

### Attitude towards EBM

The descriptive statistics of attitudes toward EBM among HCWs before and after the EBM training were presented in Table 4. On the whole, the average total score for the attitude towards EBM after training was significantly higher than before (66.5 > 63.0, *P* < 0.001). The average scores for each item of the attitude domain before and after training ranged from 2.6 to 4.5 and 2.7 to 4.5, respectively. From the descriptive statistics of the level of HCWs’ attitude towards EBM, we found that the top three levels were awarded to the statements A3, A15, and A5, while the lower three levels were A14, A10, and A6. The top three levels after training were A15, A3, and A13, while the lower three were A10, A12, and A14. In addition, HCWs obtained higher mean scores for individual statements of the attitude domain after the EBM training. There were significant differences between the mean scores of attitude towards EBM before and after training in the statements A1, A6, A7, A13, A14, and A15. Take statement A1 (I believe that evidence-based medicine is a threat to good clinical practice) for example, the mean scores of attitude towards EBM before and after training were 2.8 and 3.2, respectively.

**Table 4:** Participants’ attitude toward EBM

Item	Description	Before		After		*P*
		Mean ± sd	Level	Mean ± sd	Level	
A1	I believe that evidence-based medicine is a threat to good clinical practice.	2.8(1.4)	13	3.2(1.5)	14	**0.039**
A2	I believe practicing evidence-based medicine can improve patient health outcome.	4.1(0.7)	9	4.1(0.8)	11	0.480
A3	I am keen to learn evidence-based medicine if given the opportunity.	4.5(0.5)	1	4.5(0.7)	2	0.467
A4	I am ready to practice evidence-based medicine in my work.	4.2(0.7)	6	4.3(0.8)	6	0.141
A5	I feel that research findings are very important in my day-to-day management of patients.	4.3(0.8)	3	4.3(0.6)	7	0.395
A6	I feel that evidence-based medicine is of limited value in general practice because management in primary care requires less scientific evidence.	2.7(1.3)	15	3.4(1.0)	13	**0.001**
A7	I believe that years of clinical experience is more valuable than evidence-based medicine.	3.0(1.1)	12	3.4(1.0)	12	**0.015**
A8	I am convinced that applying evidence-based medicine in clinical practice increases the effectiveness of my work.	4.2(0.7)	8	4.3(0.6)	8	0.322
A9	I feel confident managing patients with evidence-based medicine.	3.9(0.8)	11	4.1(0.7)	10	0.154
A10	I am certain that understanding the basic mechanisms of disease is sufficient for good clinical practice.	2.7(1.2)	16	2.7(1.3)	17	0.412
A11	I feel that access to databases is vital in obtaining journals on evidence-based medicine.	4.1(0.7)	10	4.2(0.6)	9	0.180
A12	I feel that reading the conclusions of a systematic review is adequate for clinical practice.	2.8(1.2)	14	3.0(1.0)	16	0.063
A13	I feel that practicing evidence-based medicine would produce better health practitioners.	4.2(0.5)	7	4.4(0.6)	3	**0.033**
A14	I often feel burdened whenever needing to use evidence-based medicine in practice.	2.6(1.0)	17	3.2(0.9)	15	**0.002**
A15	I think it is mandatory for physicians to continuously update their knowledge to deliver efficient patient care.	4.3(0.5)	2	4.5(0.6)	1	**0.032**
A16	I am interested in receiving educational materials on evidence-based medicine as they relate to various topics.	4.3(0.5)	4	4.4(0.6)	4	0.106
A17	I think that educational interventions and incorporating formal teaching of evidence-based medicine at medical education are very important.	4.3(0.6)	5	4.4(0.6)	5	0.136
Total		63.0(5.5)		66.5(6.8)		**< 0.001**

### Practice of EBM

Table 5 reported the basic statistics of the practice of EBM among HCWs before and after the EBM training. In total, the average total scores before and after training were 34.5 and 34.7, respectively. The average scores for each statements of the practice domain before training ranged from 2.1 to 3.6. We found that the top level was awarded to the statement P7 and the mean (sd) was 3.6 (1.0). While the lower level was awarded to the statement P10, with a mean (sd) 2.1 (1.3). Similarly, the average scores of the practice domain after training ranged from 1.9 to 3.7. The top level was P7, while the lower level was P10.

**Table 5:** Participants’ practice of EBM

Item	Description	Before		After		*P*
		Mean (sd)	Level	Mean (sd)	Level	
P1	I apply evidence-based medicine in practice.	3.1(1.0)	8	3.0(1.0)	9	0.539
P2	I use multiple search engines for systematic review.	3.5(1.0)	3	3.4(1.1)	4	0.656
P3	I search for evidence-based medicine material from published journals only.	3.3(1.1)	5	3.2(1.1)	7	0.797
P4	I do not have enough time to study evidence-based medicine.	2.6(0.9)	10	2.9(0.8)	10	0.055
P5	I cannot practice evidence-based medicine due to limitations of the management that I can offer to patients in clinic settings.	3.1(0.8)	9	3.3(0.9)	6	0.052
P6	I use evidence based-medicine for answering the questions in a clinical setting.	3.2(0.9)	7	3.1(1.0)	8	0.732
P7	I join continuous medical education for an update regarding evidence-based medicine.	3.6(1.0)	1	3.7(1.0)	1	0.319
P8	I promote evidence-based practice to my colleagues at the workplace.	3.5(1.0)	2	3.5(1.0)	2	0.473
P9	I share my knowledge of evidence-based medicine with my colleagues.	3.3(1.0)	6	3.3(1.0)	5	0.359
P10	I am involved in the development of clinical practice guideline.	2.1(1.3)	11	1.9(1.2)	11	0.814
P11	I usually translate a clinical question into a form that can be answered from the literature.	3.4(1.0)	4	3.4(1.0)	3	0.375
Total		34.5(6.2)		34.7(6.9)		0.467

### Awareness of EBM resources and statistical terms

Tables 6 and 7 summarized the details of respondents’ responses toward EBM resources and statistical terms. The overall awareness of the most used EBM resources after training performed better than before training (Table 6). There was a significant difference between the two time groups, including the awareness of Bandolier, Cochrane database of systematic reviews, Best practice, Medicine (McGraw Hill), and Google Scholar. For Clinical evidence, PubMed/Medline, and Up to date, no significant difference was found. According to the survey data, except for systematic review and number needed to treat, more than 40% of the participants did not understand the common concepts such as relative risk, absolute risk, odds ratio, meta-analysis, clinical effectiveness, confidence interval, heterogeneity, and publication before the EBM training (Table 7). After the EBM training was completed, approximately more than 80% of the HCWs fully or partially understood the above concepts, which had statistical significance compared with that before the training.

**Table 6:** Participants’ awareness of EBM resources

EBM resources	Time	Unaware	Aware but not used	Read	Used to help in clinical decision-making	*P*
Bandolier	Before	50(78.1)	8(12.5)	5(7.8)	1(1.6)	**0.020**
	After	35(54.7)	19(29.7)	8(12.5)	2(3.1)	
Clinical evidence	Before	36(56.3)	18(28.1)	8(12.5)	2(3.1)	0.081
	After	27(42.2)	19(29.7)	11(17.2)	7(10.9)	
Cochrane database of systematic reviews	Before	38(59.4)	11(17.2)	9(14)	6(9.4)	**< 0.001**
	After	21(32.8)	17(26.6)	12(18.7)	14(21.9)	
Best practice	Before	44(68.8)	15(23.4)	4(6.2)	1(1.6)	**0.005**
	After	26(40.6)	23(36)	10(15.6)	5(7.8)	
PubMed/ Medline	Before	10(15.6)	2(3.1)	22(34.4)	30(46.9)	0.053
	After	3(4.7)	5(7.8)	18(28.1)	38(59.4)	
Up To Date	Before	17(26.6)	8(12.5)	19(29.7)	20(31.2)	0.147
	After	8(12.5)	12(18.7)	20(31.3)	24(37.5)	
Medicine (McGraw Hill)	Before	27(42.2)	13(20.3)	17(26.6)	7(10.9)	**0.025**
	After	13(20.3)	19(29.7)	20(31.3)	12(18.7)	
Google Scholar	Before	19(29.7)	16(25)	14(21.9)	15(23.4)	**0.006**
	After	10(15.6)	11(17.2)	25(39.1)	18(28.1)	

**Table 7:** Participants’ awareness of statistical terms

Statistical term	Time	Don’t understand	Some understanding	Understand	*P*
Relative risk	Before	34(53.1)	25(39.1)	5(7.8)	**< 0.001**
	After	12(18.7)	41(64.1)	11(17.2)	
Absolute risk	Before	39(61.0)	23(35.9)	2(3.1)	**< 0.001**
	After	13(20.3)	44(68.8)	7(10.9)	
Systematic review	Before	19(29.7)	33(51.6)	12(18.7)	**0.002**
	After	6(9.4)	39(60.9)	19(29.7)	
Odds ratio	Before	30(46.9)	24(37.5)	10(15.6)	**0.023**
	After	16(25)	35(54.7)	13(20.3)	
Meta-analysis	Before	26(40.7)	28(43.7)	10(15.6)	**< 0.001**
	After	9(14.1)	38(59.4)	17(26.5)	
Clinical effectiveness	Before	28(43.7)	30(46.9)	6(9.4)	**0.001**
	After	12(18.7)	38(59.4)	14(21.9)	
Confidence interval	Before	22(34.4)	30(46.9)	12(18.7)	**0.035**
	After	10(15.6)	39(61)	15(23.4)	
Number needed to treat	Before	29(45.3)	29(45.3)	6(9.4)	**0.004**
	After	13(20.3)	40(62.5)	11(17.2)	
Heterogeneity	Before	37(57.8)	24(37.5)	3(4.7)	**< 0.001**
	After	18(28.1)	38(59.4)	8(12.5)	
Publication bias	Before	32(50.0)	25(39.1)	7(10.9)	**0.011**
	After	18(28.1)	34(53.1)	12(18.8)	

## Discussion

EBM represents the development direction of modern medical education and has a great impact on clinical practice, health management, and medical education. Generally, previous studies have concentrated on the KAP of EBM among healthcare providers. Only a few studies focused on evaluating the performance of KAP before and after EBM training. This study evaluated the changes in KAP of HCWs before and after EBM training, and evaluated the teaching effect of EBM training through a questionnaire survey of medical staff who participated in EBM training.

Previous research has provided evidence that evidence-based healthcare teaching could lead to improvements in the knowledge, attitude, and skill of EBM [20]. This study showed a moderate level of KAP of EBM. The total average score of knowledge after EBM training was significantly higher than the score obtained before training. This could be explained that our intervention had some positive short-term effects on the participants in terms of EBM. A positive attitude is an essential requirement for HCWs, and previous research has demonstrated that positive attitudes among primary care professionals are related to better medical care delivery [21, 22]. Previous research reported that respondents with positive attitudes toward EBM accounted for a higher proportion [23, 24]. However, some studies have found unsatisfactory attitudes among the doctors who participated in their studies [25]. Similarly, this study also found that the total average score of attitude after EBM training was significantly higher than the score obtained before training. This finding was in line with the previous research that the physician who participated EBM training previously would have a significantly more positive attitude toward EBM [26]. In our study, no significant difference was found in the practice of EBM between the two-time points. This could be explained that the participants cannot change, or cannot observe if they changed, their practice in two days. In studies conducted before, there were only approximately 50% of the physicians rated their medical practices to be typically evidence-based [27, 28].

In this survey, we found that the commonly utilized EBM resources adopted by participants in clinical decision-making were “PubMed” and “Up to Date”. Our findings were consistent with the previous studies that indicated “PubMed” was the most utilized EBM resource [29, 30]. The EBM resource “Up to date” was also found as the most utilized tool in the previous research [31]. This finding, however, was different from the survey in the UK, in which “Bandolier” was found to be the most used EBM resource [32]. Although there was a significant increase in the awareness of statistical terms after EBM training, the proportion of the respondents who could understand and explain the related statistical terms to others was still low. Less than 30% of participants could understand and explain the related statistical terms to others, such as “relative risk” (17.2%), “absolute risk” (10.9%), “systematic review” (29.7%), “odds ratio” (20.3%), and “meta-analysis” (26.5%). This proportion was lower than the previous studies [30, 33]. In addition, a survey carried out among resident physicians in hospitals in Syria reported that a lower proportion of awareness of the terms “relative risk” (11.7%), “systematic review” (10.3%), “odds ratio” (6.5%), and “meta-analysis” (4.7%) [18]. These differences might be due to the diversity of HCWs responded to, as the current study participants included HCWs in a variety of job categories.

There were still some limitations that should be noticed. First, the biases associated with self-reported surveys, including subjective and exaggerated reports and recollections, cannot be ignored. Participants might provide an over or under-assessment in the questionnaire, since the study was based on their self-assessment of the survey. Second, the study sample was based on the voluntary participation of HCWs. Voluntary participation in the survey could have attracted more enthusiastic and motivated HCWs, so that the results could be more positive. In addition, the sample size was not large enough, hence, further study with a larger sample size was needed to verify the finding. Third, we did not collect data on the validity of the data collection tool. Therefore, the findings should be interpreted by keeping this important limitation in mind. Besides, considering that the data was derived from self-perceived KAP, utilizing knowledge tests and observing performance would provide more valuable findings. Fourth, this study was only based on clinical practice and did not consider any educational theories, models, or frameworks in relation to the intervention design. Fifth, considering this study was a single group pre- and post-test design, there might be a test-retest bias. Six, considering that it is difficult to observe changes in the practice of EBM in the short term, the content provided in training can only indirectly affect the practice, and large follow-up studies are needed to measure changes in EBM practice in the future. Finally, although this study explored the KAP of EBM, the barriers to EBM practice have not been elaborated. Hence, using mixed-method surveys or focused group discussions to investigate the barriers to EBM practice was recommended in future research.

## Conclusion

This study implied that EBM training may improve the knowledge and attitude of HCWs, and its teaching effect is considerable.

### Electronic supplementary material

Below is the link to the electronic supplementary material.

Supplementary Material 1

## Data Availability

The datasets generated and/or analyzed during the current study are available from the corresponding author on request.

## References

[1] Guyatt G, Claims J, Churchill D (1992). Evidence-based medicine: a new approach to teaching the practice of medicine. JAMA.

[2] Vere J, Gibson B (2019). Evidence-based medicine as science. J Eval Clin Pract.

[3] Sackett D, Rosenberg W, Gray J (1996). Evidence based medicine: what it is and what it isn’t. BMJ.

[4] Mahmić-Kaknjo M, Kadić D, Hodžić H (2015). Awareness, knowledge, use, and attitudes toward evidence based medicine in a developing country: survey of physicians in a canton in Bosnia and Herzegovina. Croat Med J.

[5] Moosavi A, Sadeghpour A, Azami-Aghdash S (2020). Evidence-based medicine among health-care workers in hospitals in Iran: a nationwide survey. J Educ Health Promot.

[6] Melnyk B, Gallagher-Ford L, Zellefrow C (2018). The first U.S. study on nurses’ evidence-based practice competencies indicates major deficits that threaten healthcare quality, safety, and patient outcomes. Worldviews Evid Based Nurs.

[7] Saunders H, Vehviläinen-Julkunen K (2018). Key considerations for selecting instruments when evaluating healthcare professionals’ evidence-based practice competencies: a discussion paper. J Adv Nurs.

[8] Norhayati M, Nawi Z (2021). Validity and reliability of the Noor evidence-based Medicine Questionnaire: a cross-sectional study. PLoS ONE.

[9] Ilic D, Maloney S (2014). Methods of teaching medical trainees evidence-based medicine: a systematic review. Med Educ.

[10] te Pas E, Wieringa-de Waard M, de Ruijter W (2015). Learning results of GP trainers in a blended learning course on EBM: a cohort study. BMC Med Educ.

[11] Thor J, Olsson D, Nordenström J (2016). The design, fate and impact of a hospital-wide training program in evidence-based medicine for physicians - an observational study. BMC Med Educ.

[12] Howard B, Diug B, Ilic D (2022). Methods of teaching evidence-based practice: a systematic review. BMC Med Educ.

[13] Çakmakkaya Ö (2021). Formal evidence-based medicine instruction in Turkish undergraduate medical education: an initial evaluation. BMC Med Educ.

[14] Stockler M, March L, Lindley R (2009). Students’ PEARLS: successfully incorporating evidence-based medicine in medical students’ clinical attachments. BMJ Evidence-Based Med.

[15] Coşkun Ö, Kıyak Y, Budakoğlu I et al. A novel approach to teach evidence-based medicine: modified PEARLS. Gazi Med J. 2022;33(4).

[16] Novack L, Jotkowitz A, Knyazer B (2006). Evidence-based medicine: assessment of knowledge of basic epidemiological and research methods among medical doctors. Postgrad Med J.

[17] Araujo G, Correia L, Siqueira J (2021). Consensus on evidence-based medicine curriculum contents for healthcare schools in Brazil. BMJ Evid Based Med.

[18] Alabdullah M, Alabdullah H, Kamel S (2022). Knowledge, attitude, and practice of evidence-based medicine among resident physicians in hospitals of Syria: a cross-sectional study. BMC Med Educ.

[19] Unadkat M, Mbuba C, Ngugi A (2021). Self-reported knowledge, attitudes, practices and barriers in use of evidence-based medicine among resident physicians in Kenya: a mixed methods study. BMC Med Educ.

[20] Young T, Rohwer A, Volmink J (2014). What are the effects of teaching evidence-based health care (EBHC)? Overview of systematic reviews. PLoS ONE.

[21] Ssekamatte T, Isunju J, Zirimala P (2021). A positive attitude among primary healthcare providers predicts better hepatitis B prevention practices: evidence from a cross-sectional survey in Wakiso district, Central Uganda. Health Psychol Behav Med.

[22] Hong J, Chen J (2019). Clinical Physicians’ attitudes towards evidence-based Medicine (EBM) and their evidence-based practice (EBP) in Wuhan, China. Int J Environ Res Public Health.

[23] Abdel-Kareem A, Kabbash I, Saied S (2019). Knowledge, practices and attitudes of physicians towards evidencebased medicine in Egypt. East Mediterr Health J.

[24] Nejašmić D, Vrdoljak D, Bralić Lang V (2020). Awareness, attitudes, barriers, and knowledge about evidence-based medicine among family physicians in Croatia: a cross-sectional study. BMC Fam Pract.

[25] Bin Briek A, Webair H, Al-Tuhaify M. Assessment of physicians’ attitude, awareness and knowledge of evidence based medicine: an observational study from Yemen. J Fam Med. 2014:1:5.

[26] Ahmadi-Abhari S, Soltani A, Hosseinpanah F (2008). Knowledge and attitudes of trainee physicians regarding evidence-based medicine: a questionnaire survey in Tehran. Iran J Eval Clin Pract.

[27] Tracy C, Dantas G, Upshur R (2003). Evidence-based medicine in primary care:qualitative study of family physicians. BMC Fam Pract.

[28] Barghouti F, Halaseh L, Said T (2009). Evidence-based medicine among Jordanian family physicians: awareness, attitude, and knowledge. Can Fam Physician.

[29] Risahmawati R, Emura S, Nishi T (2011). Japanese resident physicians’ attitudes, knowledge, and perceived barriers on the practice of evidence based medicine: a survey. BMC Res Notes.

[30] ALruwaili B, Thirunavukkarasu A, Alsaidan A (2022). Knowledge, attitude, and practice towards evidence-based medicine among Northern Saudi primary care physicians: a cross-sectional study. Healthcare.

[31] Qadhi I, AlSaidan L, AlSomali H (2021). Knowledge, attitude, practice, and barriers of evidence-based medicine among physicians in general hospitals in Kuwait: a cross-sectional study. Ann Med Surg.

[32] McColl A, Smith H, White P (1998). General practitioner’s perceptions of the route to evidence based medicine: a questionnaire survey. BMJ.

[33] Alshehri AA, Al-Khowailed MS, Alnuaymah FM (2018). Knowledge, attitude, and practice toward evidence-based medicine among hospital physicians in Qassim Region, Saudi Arabia. Int J Health Sci.

