# Supplementary material for: Self-perceived knowledge, attitude, and practice of evidence-based medicine before and after training among healthcare workers in Taizhou, China
Source: BMC Med Educ. 2024 Jun 27;24:700. doi: 10.1186/s12909-024-05678-7 (PMC11212180; doi:10.1186/s12909-024-05678-7)
Supplement: Supplementary file 1 — Supplementary Material 1 [file 12909_2024_5678_MOESM1_ESM.docx]

**Questionnaire**

The data collected by the questionnaire were only used for statistical analysis. This study was approved by the ethics committee. You are volunteered to participate in this survey.

- **Basic Information**

1. Gender: □ Male □ Female
2. Date of Birth: □□□□Year□□Month□□Day
3. Educational Level: □ Bachelor degree □ Postgraduate degree
4. Occupation: □ Doctor □ Nurse □ Medical technician □ Administration
5. Professional technical title: □ Primary grade or below □ Medium grade □ Senior grade
6. Total service years
7. Previously undergone training courses in EBM? □ Yes □ No

- **Knowledge of evidence-based medicine**

1. Knowledge of evidence-based medicine

|  |  | Strongly agree | Agree | Neutral | Disagree | Strongly disagree |
| --- | --- | --- | --- | --- | --- | --- |
| 1 | Evidence-based medicine involves the process of critically appraising research findings as to the basis for clinical decisions. | 5 | 4 | 3 | 2 | 1 |
| 2 | Evidence-based medicine focuses on the best current available research without considering clinical experience. | 1 | 2 | 3 | 4 | 5 |
| 3 | Evidence-based medicine is suitable for making decisions about the care of patients rather than for policymaking. | 1 | 2 | 3 | 4 | 5 |
| 4 | Patients’ preferences should be prioritized over clinicians’ preferences in making clinical decisions. | 1 | 2 | 3 | 4 | 5 |
| 5 | Evidence-based medicine improves clinical management by using evidence from meta-analysis only. | 1 | 2 | 3 | 4 | 5 |
| 6 | Evidence-based medicine does not help to promote self -directed learning. | 1 | 2 | 3 | 4 | 5 |
| 7 | Meta-analysis is superior to case-control studies in evidence-based medicine. | 5 | 4 | 3 | 2 | 1 |
| 8 | Four essential components structured in the PICO format (Patient or problem, Intervention, Comparison, Outcome) will make a good clinical question. | 5 | 4 | 3 | 2 | 1 |
| 9 | Evidence-based medicine improves clinicians’ understanding of research methodology. | 5 | 4 | 3 | 2 | 1 |
| 10 | Clinicians who practice evidence-based medicine become less critical in using data in systemic reviews. | 1 | 2 | 3 | 4 | 5 |
| 11 | Evidence-based medicine can be practiced in situations where there is doubt about any aspect of clinical management. | 5 | 4 | 3 | 2 | 1 |
| 12 | Improving access to summaries of evidence is appropriate to encourage evidence-based practice. | 5 | 4 | 3 | 2 | 1 |
| 13 | The increasing number of systematic reviews that are applicable to general practice can be found in the Cochrane Library. | 5 | 4 | 3 | 2 | 1 |
| 14 | Difficulty in understanding statistical terms is the major setback in applying evidence-based medicine. | 5 | 4 | 3 | 2 | 1 |
| 15 | Application of evidence-based practice is cost-effective to the healthcare system. | 5 | 4 | 3 | 2 | 1 |

- **Attitude on evidence-based medicine**

1. Attitude on evidence-based medicine

|  |  | Strongly agree | Agree | Neutral | Disagree | Strongly disagree |
| --- | --- | --- | --- | --- | --- | --- |
| 1 | I believe that evidence-based medicine is a threat to good clinical practice. | 1 | 2 | 3 | 4 | 5 |
| 2 | I believe practicing evidence-based medicine can improve patient health outcome. | 5 | 4 | 3 | 2 | 1 |
| 3 | I am keen to learn evidence-based medicine if given the opportunity. | 5 | 4 | 3 | 2 | 1 |
| 4 | I am ready to practice evidence-based medicine in my work. | 5 | 4 | 3 | 2 | 1 |
| 5 | I feel that research findings are very important in my day-to-day management of patients. | 5 | 4 | 3 | 2 | 1 |
| 6 | I feel that evidence-based medicine is of limited value in general practice because management in primary care requires less scientific evidence. | 1 | 2 | 3 | 4 | 5 |
| 7 | I believe that years of clinical experience is more valuable than evidence-based medicine. | 1 | 2 | 3 | 4 | 5 |
| 8 | I am convinced that applying evidence-based medicine in clinical practice increases the effectiveness of my work. | 5 | 4 | 3 | 2 | 1 |
| 9 | I feel confident managing patients with evidence-based medicine. | 5 | 4 | 3 | 2 | 1 |
| 10 | I am certain that understanding the basic mechanisms of disease is sufficient for good clinical practice. | 1 | 2 | 3 | 4 | 5 |
| 11 | I feel that access to databases is vital in obtaining journals on evidence-based medicine. | 5 | 4 | 3 | 2 | 1 |
| 12 | I feel that reading the conclusions of a systematic review is adequate for clinical practice. | 1 | 2 | 3 | 4 | 5 |
| 13 | I feel that practicing evidence-based medicine would produce better health practitioners. | 5 | 4 | 3 | 2 | 1 |
| 14 | I often feel burdened whenever needing to use evidence-based medicine in practice. | 1 | 2 | 3 | 4 | 5 |
| 15 | I think it is mandatory for physicians to continuously update their knowledge to deliver efficient patient care. | 5 | 4 | 3 | 2 | 1 |
| 16 | I am interested in receiving educational materials on evidence-based medicine as they relate to various topics. | 5 | 4 | 3 | 2 | 1 |
| 17 | I think that educational interventions and incorporating formal teaching of evidence-based medicine at medical education are very important. | 5 | 4 | 3 | 2 | 1 |

- **Practice of evidence-based medicine**

1. Practice of evidence-based medicine

|  |  | Always | Often | Sometimes | Seldom | Never |
| --- | --- | --- | --- | --- | --- | --- |
| 1 | I apply evidence-based medicine in practice. | 5 | 4 | 3 | 2 | 1 |
| 2 | I use multiple search engines for systematic review. | 5 | 4 | 3 | 2 | 1 |
| 3 | I search for evidence-based medicine material from published journals only. | 5 | 4 | 3 | 2 | 1 |
| 4 | I do not have enough time to study evidence-based medicine. | 1 | 2 | 3 | 4 | 5 |
| 5 | I cannot practice evidence-based medicine due to limitations of the management that I can offer to patients in clinic settings. | 1 | 2 | 3 | 4 | 5 |
| 6 | I use evidence based-medicine for answering the questions in a clinical setting. | 5 | 4 | 3 | 2 | 1 |
| 7 | I join continuous medical education for an update regarding evidence-based medicine. | 5 | 4 | 3 | 2 | 1 |
| 8 | I promote evidence-based practice to my colleagues at the workplace. | 5 | 4 | 3 | 2 | 1 |
| 9 | I share my knowledge of evidence-based medicine with my colleagues. | 5 | 4 | 3 | 2 | 1 |
| 10 | I am involved in the development of clinical practice guideline. | 5 | 4 | 3 | 2 | 1 |
| 11 | I usually translate a clinical question into a form that can be answered from the literature. | 5 | 4 | 3 | 2 | 1 |

- **EBM resources**

1. Below is a list of EBM resources most used by resident physicians. Please indicate those which you have used or are aware of.

|  |  | Unaware | Aware but not used | Read | Used to help in clinical decision‑making |
| --- | --- | --- | --- | --- | --- |
| 1 | Bandolier |  |  |  |  |
| 2 | Clinical Evidence |  |  |  |  |
| 3 | Cochrane database of Systematic Reviews |  |  |  |  |
| 4 | Best Practice |  |  |  |  |
| 5 | PubMed/Medline |  |  |  |  |
| 6 | UpToDate |  |  |  |  |
| 7 | Medicine (McGraw Hill) |  |  |  |  |
| 8 | Google Scholar |  |  |  |  |

- **Technical terms**

1. Below is a list of some of the common EBM terms used, please describe your level of understanding of each:

|  |  | Don’t understand | Some understanding | Understood |
| --- | --- | --- | --- | --- |
| 1 | Relative risk |  |  |  |
| 2 | Absolute risk |  |  |  |
| 3 | Systematic review |  |  |  |
| 4 | Odds ratio |  |  |  |
| 5 | Meta‑analysis |  |  |  |
| 6 | Clinical effectiveness |  |  |  |
| 7 | Confidence interval |  |  |  |
| 8 | Number needed to treat |  |  |  |
| 9 | Heterogeneity |  |  |  |
| 10 | Publication bias |  |  |  |
